# Bone Regeneration in Vertical Osseous Defect in Periodontitis Patients using Bovine Colostrum

**DOI:** 10.1155/2022/4183552

**Published:** 2022-06-21

**Authors:** P. L. Ravishankar, Preethikaa Guruprasath, Padmaja Vangipuram, Jasmine Vaidya, G. Visithiriyan, A. Thirumal Raj, Shilpa Bhandi, Shankargouda Patil, Snehashish Ghosh

**Affiliations:** ^1^Department of Periodontics, SRM Kattankulathur Dental College, Potheri, 603203, Tamil Nadu, India; ^2^Department of Oral Pathology and Microbiology, Sri Venkateswara Dental College and Hospital, Chennai 600130, India; ^3^Department of Restorative Dental Science, Division of Operative Dentistry, College of Dentistry, Jazan University, Jazan 45142, Saudi Arabia; ^4^Department of Maxillofacial Surgery and Diagnostic Sciences, Division of Oral Pathology, College of Dentistry, Jazan University, Jazan 45142, Saudi Arabia; ^5^Department of Oral Pathology, College of Medical Sciences, Bharatpur, Nepal

## Abstract

**Background:**

The treatment plan for periodontitis may include both nonsurgical and surgical phases. During surgical procedures, bone grafts and barrier membranes were used after degranulation in order to achieve healing. Colostrum is one of the materials that is composed of bioactive components which has either osteoinductive or regenerative potential.

**Aim:**

The aim of the present study is to evaluate the effectiveness of bovine colostrum as bone regeneration material in periodontitis. *Case Description*. Clinical periodontal parameters, including probing depth (PD), clinical attachment level (CAL), bleeding on probing (BOP), and plaque index (PI), were evaluated. Patients who were diagnosed with localised periodontitis were selected. Three patients presenting vertical defect at buccal sites were treated with bovine colostrum. Following nonsurgical treatment, flap surgery was performed using bovine colostrum. After 6 months, favourable clinical and radiographical improvements were obtained.

**Conclusion:**

All cases showed reduction in PD; these findings suggest that the bovine colostrum could favour periodontal regeneration. The clinical significance is that bovine colostrum is cost effective and easily available and enhances bone regeneration. It can therefore be used as an alternative to bone grafts during periodontal surgery.

## 1. Introduction

Periodontal disease can be localised or generalised. Unlike common perception, periodontitis cases are not restricted to the elderly population [1]. Cases have been recorded at adolescence and are characterised by interproximal attachment loss of the first molar and/or incisors, absence of inflammation, a deep probing depth, and progressive bone resorption. In many cases, plaque is insignificant in comparison to the amount of damage it causes, which can be largely attributed to the bacteria such as *Aggregatibacter actinomycetemcomitans* (*A.a*) and *Porphyromonas gingivalis* (*P.g*). Secondary clinical symptoms include diastema development between incisors with distolabial migration, mobility of the affected teeth, sensitivity of the denuded root, deep dull radiating jaw pain, and periodontal abscess lymph node enlargement [2].

In younger patients with a higher rate of bone loss, a well-planned treatment strategy and, in some cases, a more intensive treatment approach are required to prevent future periodontal deterioration and restore as much periodontal attachment as feasible [3]. Periodontal surgical therapy allows for access to bone abnormalities, allowing for osseous resection, recontouring, and other procedures such as regeneration. To rebuild the periodontal structures that have been destroyed, many regenerative procedures like bone grafting, GTR, and tissue engineering have been used [1].

The initial milk produced after the birth of the calf is known as bovine colostrum (BC). BC is necessary for the new-born calf's nutritional and immunological support, growth, and development. It is made by the dairy industry in powder form, and it is offered commercially to potentially aid in health and immunological support. This powder is mixed with water and consumed orally. It has been shown to have bioactive components including immunoglobulins and growth factors, as well as oligosaccharides, antimicrobials, and immune-regulating factors [4].

Although bone grafts are considered an ideal material for regeneration, they are relatively expensive. In contrary, bovine colostrum is cost-effective and has been effectively used postendodontic surgery to treat periapical defects [5]. Thus, bovine colostrum powder (immune strong, B colostrum, an Ayurveda proprietary drug from India) was employed during periodontal surgery to fill the bone deficiency in the present case series. The present case series was aimed at evaluating the effectiveness of bovine colostrum as bone regeneration material in vertical osseous defect of periodontitis patients in a relatively younger age group.

## 2. Case Description

### 2.1. Case 1

**Figure 1:**
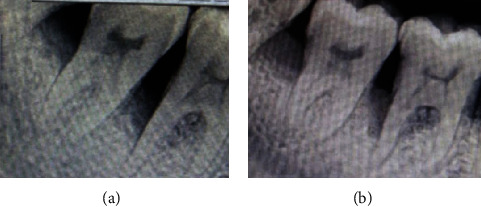
Radiographic image: (a) preoperative and (b) postoperative.

**Figure 2:**
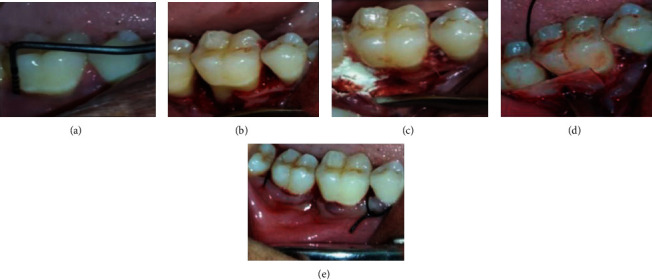
(a) PPD-10 mm. (b) Debridement done. (c) Placement of bovine colostrum. (d) GTR placement—suturing done.

A 32-year-old female patient visited our department with complaint of pain in the right lower back tooth region for the past 2 months. The patient was in good health. The patient has undergone extraction before 3 months. In respect to the mandibular right first molar, probing depth of 10 mm, grade II tooth mobility, suppuration and bleeding on probing, and gingival irritation were all noted. In relation to 46, a periapical radiograph was taken (Figure 1). Radiolucency extending from middle one-third of the distal aspect of the root of 46 to middle one-third of the root of the mesial aspect of the root of 47 is suggestive of vertical bone defect. Periodontal damage was manifested by deep infrabony deformities. The patient got root planing at 1-week intervals after full-mouth scaling. Systemic antibiotics were recommended. After three weeks of phase I therapy, the flap surgery was performed. A full-thickness mucoperiosteal flap was reflected with sulcular incision and interdental incision from the distal aspect of 45 till the mesial aspect of 47. Debridement of granulation tissue was done followed by sterile saline irrigation. The required amount of bovine colostrum (immune strong, B colostrum, an Ayurveda proprietary drug from India) was taken in a sterile Dappen dish and adapted to the defect in powder form. The GTR membrane was placed, and suturing was done using 3-0 silk thread (Figure 2). After the surgery, the patient was given postoperative instructions and medications. At 3 months, clinical measures were taken with a manual periodontal probe. Clinical benefits were shown in measurements. The afflicted areas' PD has dropped to a maximum of 4 mm. Six months after therapy, radiographs revealed that the bone defects had been filled (Figure 2).

### 2.2. Case 2

**Figure 3:**
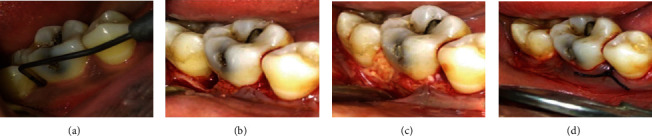
(a) PPD-10 mm. (b) Debridement done. (c) Placement of bovine colostrum. (d) Suturing done.

A 28-year-old male patient visited our department with complaint of mobile teeth. The patient was in good health. The patient had dental history of restoration done before 5 months. In respect to the mandibular right first molar, probing depth of 11 mm, grade II tooth mobility, and the presence of suppuration and bleeding on probing and gingival inflammation were noted. A periapical radiograph in relation to 46 was taken. Deep infrabony defects were observed in relation to 46. The patient got root planing at 1-week intervals after full-mouth scaling. Systemic antibiotics were recommended. After three weeks of phase I therapy, the flap surgery was performed. A full-thickness mucoperiosteal flap was reflected with sulcular incision and interdental incision from the distal aspect of 45 till the mesial aspect of 47. Debridement of granulation tissue was done followed by sterile saline irrigation. The required amount of bovine colostrum was taken in a sterile Dappen dish, mixed with saline and made into dough form, and adapted to the defect. The GTR membrane was placed, and suturing was done using 3-0 silk thread (Figure 3). After the surgery, the patient was given postoperative instructions and medications. The patient did not report back for review.

### 2.3. Case 3

**Figure 4:**
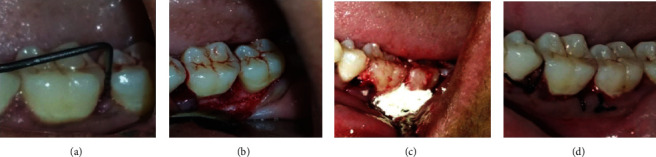
(a) PPD-10 mm. (b) Debridement done. (c) Placement of bovine colostrum. (d) Suturing done.

**Figure 5:**
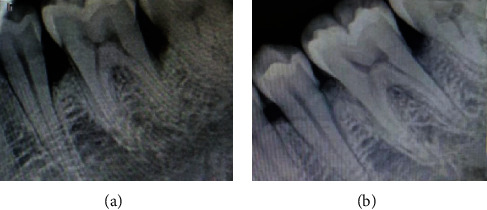
Radiographic image: (a) preoperative and (b) postoperative.

A 35-year-old female patient visited our department for regular dental check-up. The patient had medical history of hypothyroidism, and she was under medication for the past three years. This was the patient's first visit to the dentist. In respect to the mandibular left first molar, PD of 9 mm, grade I tooth mobility, bleeding on probing, and gingival inflammation were noted. A periapical radiograph in relation to 36 was taken. Radiolucency extending from the distal aspect of the root of 35 to the mesial aspect of the root of 36 and also extending from the distal aspect of the root of 36 to the mesial aspect of the root of 37 is suggestive of vertical bone defect. Deep infrabony defects were observed in relation to 35 and 36. The patient got root planing at 1-week intervals after full-mouth scaling. Systemic antibiotics were recommended. After three weeks of phase I therapy, the flap surgery was performed. A full-thickness mucoperiosteal flap was reflected with sulcular incision and interdental incision from the distal aspect of 35 till the mesial aspect of 37. Debridement of granulation tissue was done followed by sterile saline irrigation. The required amount of bovine colostrum was taken in a sterile Dappen dish and adapted to the defect in powder form. The GTR membrane was placed, and suturing was done using 3-0 silk thread (Figure 4). After the surgery, the patient was given postoperative instructions and medications. At 3 months, clinical measures were taken with a manual periodontal probe. Clinical benefits were shown in measurements. The afflicted areas' PD has dropped to a maximum of 4 mm. Six months after therapy, radiographs revealed that the bone defects had been filled (Figure 5).

## 3. Discussion

Periodontal disease is an inflammatory disorder that affects the tissues around the teeth as a result of dental plaque on the surface of the teeth. The breakdown of supporting tissues occurs as a result of the host-activated inflammatory response, which is usually triggered by gram-negative microorganisms. Several regeneration approaches have been examined as a supplement to surgical therapy [6, 7].

Bovine colostrum is a nutritional supplement, and it has been shown that colostrum in oral care products boosts the natural defence system and improves periodontal repair [4]. Other clinical uses of bovine colostrum include improvement of oral hygiene in people with oral lichen planus and Sjogren's syndrome [8], dietary supplements in immunodeficiency-related diarrhoea, prophylaxis and treatment of infectious diseases caused by *Vibrio cholerae*, *Helicobacter pylori*, and rotavirus, and treatment of multiple sclerosis and gastrointestinal inflammation caused by nonsteroidal anti-inflammatory drugs [9, 10].

Lactose, oligosaccharides, glycolipids, glycoproteins, and nucleotide sugars are all carbohydrates found in BC. It also contains about 7% fat, which is mostly made up of milk fat globules. BC is high in water-soluble vitamins and fat-soluble, both of which are important for a variety of biological activities, especially bone formation and antioxidant properties. Improved immune function and mental health have also been related to vitamin D [9, 11].

Colostrinin (also known as PRP or proline-rich polypeptide) is a naturally present blend of proline-rich polypeptides that have been demonstrated to assist in the reduction of excessive inflammatory responses. It contains Milk Fat Globule-Epidermal Growth Factor 8 (MFG-E8) and also growth factors such as insulin-like growth factor, epidermal growth factor, transforming growth factor, platelet-derived growth factor, and vascular endothelial growth factor. MFG-E8 is a secreted protein that was shown to be a key part of the bovine colostrum milk. It improves phagocytosis' clearance of injured and apoptotic cells, stimulation of VEGF-mediated new vessel creation, and mucosal repair [4, 9–11].

An effective amount of bovine colostrum, according to the invention, has antibacterial or antimicrobial action in the oral cavity. Immunoglobulins, growth factors, lactoferrin, lysozyme, lactoperoxidase, cytokines and nucleosides, calcium, and phosphate are among the bioactive compounds found in it [5, 8, 12]. Bovine colostrum has been employed in periapical surgery and had achieved bone gain without any side effects [5]. Thus, based on the clinical success evidenced in the published literature, bovine colostrum powder was used in the current case series to fill the osseous defect.

Cases 1 and 3 had remarkable surgical healing. Clinical improvements were observed six months after treatment. The PD reduction was between 3 and 4 mm. There was no longer any suppuration, and there were no symptoms of gingival inflammation. The periapical radiographs taken 6 months following therapy revealed that the bone deficiencies had been filled and that the treatment outcome had remained stable. Although all sites showed similar clinical benefits, there were differences in the degree of radiological defect filling. This finding was in contrary to Veeramachaneni et al. [5] where bovine colostrum was used in the management of periapical lesion.

The bovine colostrum was used in powder form, and this may help in faster resorption of the material. This was in contrary to the study done by Bhaskar et al. [13] where they reported that larger particle size required prolonged time for resorption. Prior to therapy with bovine colostrum, a periapical radiograph was done to examine the bone defect. Colostrum appeared radiolucent at first, and the radiopacity gradually increased over time. This emphasises colostrum's ability to promote regeneration.

When compared to alternative bone grafts, bovine colostrum is less expensive. Growth factors included in bovine colostrum, including transforming growth factor, insulin-like growth factor, and epidermal growth factor, have been shown to aid in the regeneration and repair of numerous bodily tissues [5]. These same elements could have had a role in bone healing in these circumstances.

## 4. Conclusion

Based on the clinical and radiological findings of the 3 cases included in the present case series, there is preliminary evidence to suggest that colostrum-based treatment of bone deficiency following periodontal surgery had a beneficial effect. There are a few limitations in this case series. These include a lack of microbiological and histological evaluation. In addition, being a treatment-based report, a case series with 3 cases would not be sufficient to objectively assess the success of the treatment. Thus, considering the present case series as preliminary evidence, further large-scale multicentre randomized controlled trials are needed to validate the findings of the present report.

## Data Availability

Research data are not shared.
